# Spasmolytic Activity of *Gentiana lutea* L. Root Extracts on the Rat Ileum: Underlying Mechanisms of Action

**DOI:** 10.3390/plants13030453

**Published:** 2024-02-04

**Authors:** Nemanja Kitić, Jelena Živković, Katarina Šavikin, Milica Randjelović, Miloš Jovanović, Dušanka Kitić, Bojana Miladinović, Milica Milutinović, Nenad Stojiljković, Suzana Branković

**Affiliations:** 1 Faculty of Medicine, Research Centre for Biomedicine, University of Niš, Ave. Dr. Zorana Đinđića 81, 18000 Niš, Serbia; 2 Institute for Medicinal Plants Research “Dr. Josif Pančić”, Tadeuša Košćuška 1, 11000 Belgrade, Serbia; jzivkovic@mocbilja.rs (J.Ž.); ksavikin@mocbilja.rs (K.Š.); 3 Faculty of Medicine, Department of Pharmacy, University of Niš, Ave. Dr. Zorana Đinđića 81, 18000 Niš, Serbia; milica.randjelovic@medfak.ni.ac.rs (M.R.); milos.jovanovic@medfak.ni.ac.rs (M.J.); bojana.miladinovic@medfak.ni.ac.rs (B.M.); milica.milutinovic@medfak.ni.ac.rs (M.M.); 4 Faculty of Medicine, Department of Physiology, University of Niš, Ave. Dr. Zorana Đinđića 81, 18000 Niš, Serbia; nenstojiljkovic@gmail.com (N.S.); brankovic.suzana@yahoo.com (S.B.)

**Keywords:** *Gentiana lutea* L., extracts, fractionization, spasmolytic, ileum, rats

## Abstract

The roots of *Gentiana lutea* L. are utilized in the preparation of various beverages and herbal remedies, serving as a traditional remedy for gastrointestinal ailments. The spasmolytic activity that could substantiate the traditional use of *G. lutea* root had not been investigated. The main objective goal of the study was to determine the validity of its use as a traditional remedy. The extraction of *G. lutea* root was performed using a 50% hydroethanolic solvent with three different extraction techniques: ultrasound-assisted extraction (UAE), heat-assisted extraction, and percolation. The spasmolytic activity was tested on isolated rat ileum. The mechanism of action was monitored using the models of spontaneous contractions and acetylcholine-, histamine-, CaCl_2_-, Bay K8644-, L-NAME-, ODQ-, apamin-, BaCl_2_-, charybdotoxin-, glibenclamide-, TRAM-34-, and quinine-modified contractions. UAE, having the best bioactivity, was further subjected to a liquid–liquid extraction fractionation. HPLC phytochemical analysis was performed for all tested extracts and fractions. Gentian root extracts were rich in secoiridoids, xanthones, and flavonoids. The UAE has shown better results on spontaneous contractions in comparison to its fractions, leading to the more detailed testing of its spasmolytic mechanism of activity. The extract’s activity is primarily mediated through intermediate conductance Ca^2+^-activated K^+^ channels, ATP-sensitive K^+^ channels, voltage-sensitive K^+^ channels, and mechanisms that activate Ca^2+^ channels. Overall, the *G. lutea* root shows great potential in the treatment of spasmodic gastrointestinal ailments.

## 1. Introduction

Traditional medicine, also known as folk medicine, is widely used across the world. According to some accounts, up to 80% of the population in some world regions use traditional medicine as a primary source of health care [1]. Plants with healing properties, as an indisputable foundation of traditional medicine, have started to become more prevalent in modern medicine because of new extraction and processing methodologies [2]. Different plant-based extracts have shown a variety of health benefits such as anti-inflammatory, anti-cancer, anti-oxidative properties, and more. These extracts, which strive to fix imbalances in the body, are used actively in the treatment of many diseases as both a supplement and main therapy [3].

Disruptions in the motility of the ileum smooth muscle can contribute to functional issues within the gastrointestinal tract, potentially leading to conditions such as irritable bowel syndrome and dyspepsia. These imbalances may be followed by obstipation, diarrhea, and stomach pains [4]. Plant-based products, as used in both modern and traditional medicine, can reduce symptoms of heightened muscle contractions of the gastrointestinal system. These products affect the smooth muscles directly or by releasing neurotransmitters from the enteric nervous system [5].

The Gentianaceae family comprises over 400 plant species that are spread across the world from the Alps through South and West Europe, the Americas, the Australian continent, and the Pyrenees, sometimes even up to heights of 2500 m [6]. Within this family, plants from the genus *Gentiana* L., with nearly 600 isolated metabolites and around 20 confirmed bioactivities, are widely recognized as herbal remedies with a long history [7]. The best-known species of this genus, and the one listed in the European Pharmacopoeia, is *Gentiana lutea* L., commonly known as yellow gentian. It is a perennial plant with a strong stem from which large leaves grow near the ground. The fruit is a capsule, and its leaves grow at the top of the plant. They are a golden-yellow color, and they are combined with small leaves that grow alongside them. The underground parts are quite developed. Thick roots grow from the rhizome, which is short and forked. The raw root is very soft and white inside and has a very bitter taste [8,9].

The roots of *G. lutea* are utilized in preparing various beverages and herbal remedies, serving as a traditional remedy for gastrointestinal ailments, especially for appetite loss and flatulence [10]. Extracts made from the plant stimulate the secretion of stomach acid [10]. They are also used for convulsions and colds and in treating gastritis, jaundice, and other gastrointestinal and hepatic diseases [11]. Low doses of the drug stimulate the nervous system, while high doses have a sedating effect [12]. The medicinal properties of the roots are primarily attributed to the presence of bitter compounds, such as secoiridoids such as gentiopicroside, swertiamarin, and sweroside, as well as biphenyl secoiridoid derivatives like amarogentin, recognized as one of the most common, naturally occurring, bitter compounds [6,13]. Besides secoiridoides, the roots contain various phytocompounds with well-documented bioactivities, including xanthones such as isogentisin, gentisin, and gentioside, iridoid loganic acid, as well as phenolics and miscellaneous compounds [14]. Confirmed pharmacological activities suggest the potential of *G. lutea* preparations for liver [15], cardiovascular [16], skin [17], and reproductive protection [18].

To the best of our knowledge, the spasmolytic activity that could substantiate the traditional use of *G. lutea* root, particularly in the context of gastrointestinal disorders, has not been thoroughly investigated. Some representatives of this genus, such as *Gentiana spathacea* Kunth [19], have shown spasmolytic activities; however, a comprehensive understanding of the underlying mechanism of action remains unresolved.

Despite significant progress in developing advanced techniques for extracting bioactive compounds from plant material, these methods are often costly, lack well-established safety, and require sophisticated equipment. Therefore, conventional techniques remain widely used due to their perceived safety and cost-effectiveness [20]. To efficiently extract bioactive compounds from *G. lutea* root, traditional solid–liquid extraction using an aqueous–alcoholic mixture can be effectively employed [14]. Extraction conditions can significantly influence the phytochemical composition, as evidenced by numerous studies, yet there is considerably less available data on their expected impact on bioactivities. Given the rising demand for plant-based natural products in drug, nutraceutical, and cosmetics development, contemporary research in this field emphasizes the crucial task of establishing the most effective extraction methods and optimizing them tailored to each raw material, taking into account the specific features of target compounds [2]. Since the extraction process may result in the co-extraction of compounds such as sugars or dietary fibers alongside the desired bioactive phytochemicals, it is necessary to further fractionate the crude extracts [20].

The main objective of this work was to investigate gastrointestinal spasmolytic properties of *G. lutea* root extracts obtained through various extraction techniques, including conventional heat-assisted extraction, triple percolation, and accelerated ultrasound-assisted extraction. Furthermore, the most promising extract underwent a fractionation using liquid–liquid extraction, and the obtained fractions were subjected to comprehensive analysis to elucidate the underlying mechanism of action and establish correlations between activities and phytochemical composition.

In the present work, the spasmolytic activities of *G. lutea* root extracts, with a narrow focus on their mechanism of action, were studied for the first time. The results presented in this study contribute to the understanding of the pharmacological activities of this plant, which is highly valued in traditional medicine, establishing a foundation for its application in rational phytotherapy for gastrointestinal disorders.

## 2. Results

### 2.1. Chemical Composition of G. lutea Root Extracts and Fractions

In crude extracts obtained through an ultrasound-assisted extraction, a heat-assisted extraction, and percolation, six individual secondary metabolites belonging to the classes of iridoids and secoiridoids (loganic acid, sweroside, swertiamarin, and gentiopicroside), xanthones (isogentisin), and flavonoids (isovitexin) were quantified (Table 1). UAE had the highest extraction efficiency for all individual compounds, except for gentiopicroside, where the highest concentration was recorded in the case of percolation. All HPLC chromatograms are represented in the supplementary files (Figures S1–S7).

Considering UAE’s high extraction efficiency, its list of compounds, and the results of the spasmolytic activity against spontaneous rat ileum contractions, crude UAE was selected as the most suitable for subsequent fractionation. Fractionation was carried out using liquid–liquid extraction employing a series of organic solvents with increasing polarity, and the recorded results of the chemical analysis are presented in Table 1. As expected, fractionation resulted in a change in the phytochemical profile compared to the initial crude extract. In the petroleum ether and ethyl acetate fractions, isogentisin was predominant (262.30 ± 5.03 and 871.58 ± 9.16 mg/g DW, respectively), while in the n-butanol and water fractions, gentiopicroside was the dominant compound (221.71 ± 8.87 and 16.07 ± 0.51 mg/g DW, respectively). Regarding observed individual iridoid and secoiridoid compounds, there are some discrepancies in the distribution pattern, probably due to their difference in solubility. Loganic acid and sweroside showed the highest separation affinity for the ethyl acetate fraction (133.22 ± 4.08 and 62.95 ± 1.27 mg/g DW, respectively). On the other hand, gentiopicroside and swertiamarin were found in the highest concentrations in the n-butanol fraction (15.54 ± 0.61 and 221.71 ± 8.87 mg/g DW, respectively). The xanthone isogentisin was completely separated in organic fractions, with a trend of contents in the order of ethyl acetate > petroleum ether > n-butanol fraction, so it was not detected in the final water fraction. Unambiguously, the re-extraction of isogentisin is favored by less polar organic solvents.

### 2.2. Spasmolytic Activity

#### 2.2.1. Effects of *G. lutea* Extracts and Fractions on Spontaneous Ileum Contractions

The examined *G. lutea* extracts demonstrated a noteworthy relaxation of ileum smooth muscle spontaneous contractions in a manner dependent on the administered dosage (0.005–5 mg/mL). Spasmolytic activity was measured for three different extracts of *G. lutea* among which the extract with the most significant effect (UAE) was further investigated and fractionated. The extracts achieved a statistically significant inhibitory effect (*p* < 0.05) on the isolated rat ileum. However, although the spasmolytic effects of the extracts were significant, they were comparatively weaker in intensity compared to the non-specific myorelaxant, papaverine.

**Figure 1:**
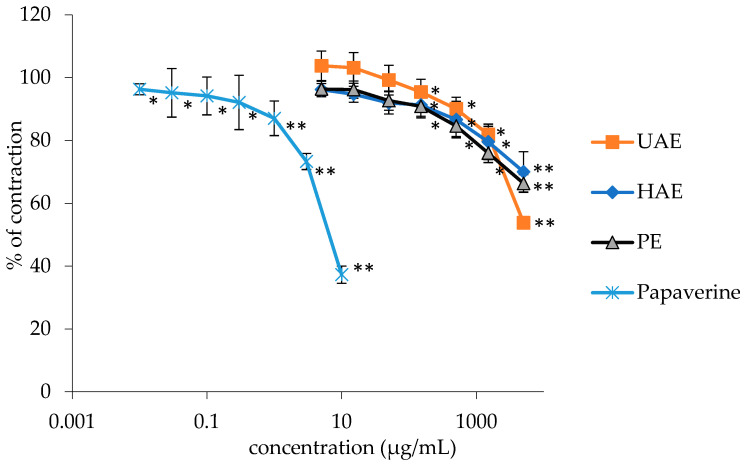
Relaxant effects of the *G. lutea* L. extracts and papaverine on spontaneous rat’s ileum contractions. Each data point represents the mean value of contractions in relation to contractions observed in the Tyrode solution (control), accompanied by the SD of six segments. Statistical analysis was conducted using Student’s *t*-test, with * *p* < 0.05 and ** *p* < 0.01 denoting significance compared to the Tyrode solution. UAE, HAE, PE—extract obtained by ultrasound-assisted extraction, heat-assisted extraction, and percolation.

Papaverine, at a maximum applied concentration of 0.01 mg/mL, inhibited 62.72 ± 2.72% of contractions out of 100%, whereas the extracts, at a maximum concentration of 5 mg/mL, reduced contractions in the range of 29.98 ± 2.56 to 46.19 ± 4.60%. Table 2 shows the EC_50_ values and maximum contraction inhibition of all extracts of *G. lutea*. Additionally, Figure 1 depict the dose-dependence of spasmolytic activity of the tested extracts and papaverine.

**Figure 2:**
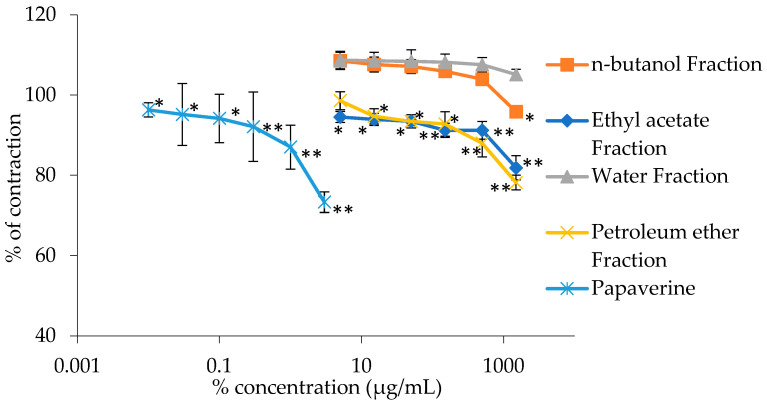
Relaxant effects of the *G. lutea* L. fractions and papaverine on spontaneous rat’s ileum contractions. Each data point represents the mean value of contractions in relation to contractions observed in the Tyrode solution (control), accompanied by the SD of six segments. Statistical analysis was conducted using Student’s *t*-test, with * *p* < 0.05 and ** *p* < 0.01 denoting significance compared to the Tyrode solution.

The extract that showed the best impact on spontaneous contractions, UAE, was fractionized into separate fractions, whose impact on spontaneous contractions was also examined (Figure 2). The best fraction was the petroleum ether fraction that reduced the spontaneous ileum contractions by 32.15 ± 2.08% when given in its highest concentration of 1.5 mg/m. The other fraction that showed a great response was the ethyl acetate fraction that reduced the contractions by 28.77 ± 2.62%. The other two fractions, n-butanol and water fractions, showed very low to no inhibitory values.

Given that the best inhibition of spontaneous contractions was achieved with the UAE, further investigations were carried out with that extract.

#### 2.2.2. Effects of the *G. lutea* Extract on Acetylcholine-Induced Ileum Contractions

**Figure 3:**
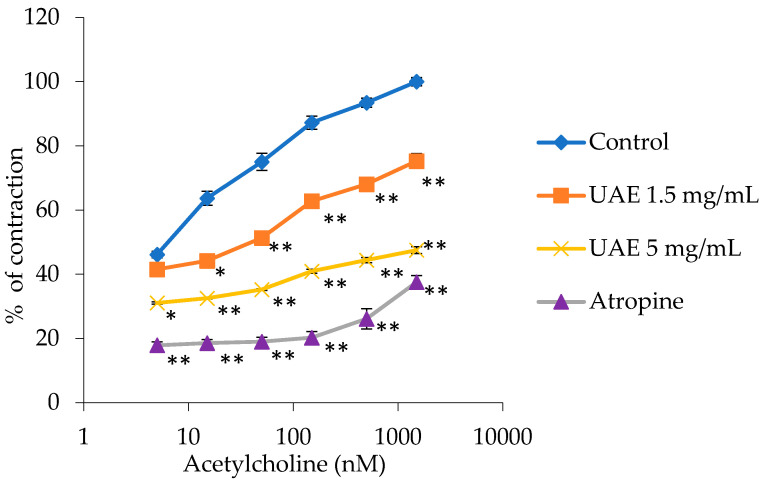
Relaxant effects of the *G. lutea* L. extract, UAE (1.5 mg/mL and 5 mg/mL), and atropine (140 nM) on the acetylcholine-induced contractions of the isolated rat’s ileum. Each data point represents the mean value of the response, accompanied by the SD of six segments. Statistical analysis was carried out using Student’s *t*-test, with * *p* < 0.05 and ** *p* < 0.01 indicating significance compared to the control. UAE—extract obtained by ultrasound-assisted extraction.

The utilization of *G. lutea* UAE extract resulted in a substantial reduction in ileum contractions triggered by cumulative doses of acetylcholine solution (5–1500 nM). This effect was evident in the alteration of the effective concentration (EC_50_) of acetylcholine, as illustrated in Figure 3.

The addition of the extract at concentrations of 1.5 mg/mL and 5 mg/mL resulted in an elevation of the EC_50_ values for acetylcholine (control), suggesting a decrease in induced contractions. The extract altered the control EC_50_ value of acetylcholine from 39.85 ± 0.19 nM to 85.32 ± 2.56 nM with the 1.5 mg/mL extract concentration and to 120.44 ± 1.19 nM with the 5 mg/mL extract concentration. Contractions at 100%, induced by the maximum dose of acetylcholine at 1500 nM, were reduced to 75.29 ± 2.30% with 1.5 mg/mL and to 47.53 ± 1.05% with 5 mg/mL of this extract. 

Atropine, used as a positive control, modified the EC_50_ value of acetylcholine from 39.85 ± 0.19 nM to 480 ± 11.64 nM and reduced the maximum ileum contraction (100%) to 37.55 ± 2.07%.

#### 2.2.3. Effects of the *G. lutea* Extract on Histamine-Induced Ileum Contractions

**Figure 4:**
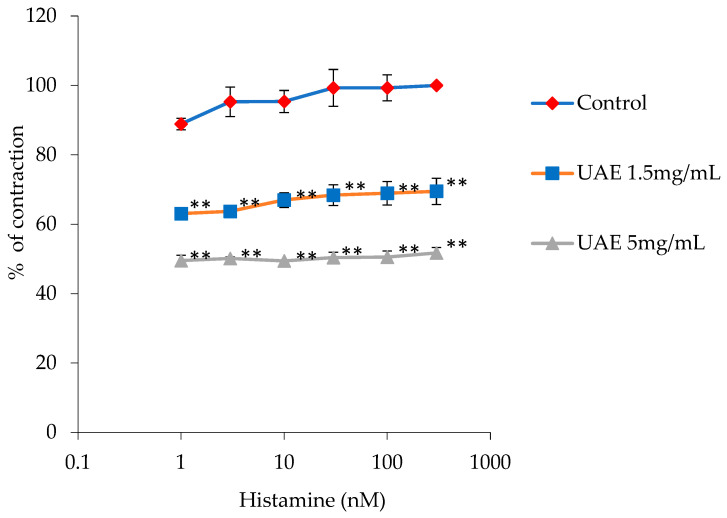
Relaxant effects of the *G. lutea* L. extract, UAE (1.5 mg/mL and 5 mg/mL), on the histamine-induced contractions of the isolated rat’s ileum. Each data point represents the mean value of the response, accompanied by the SD of six segments. Statistical analysis was carried out using Student’s *t*-test, with ** *p* < 0.01 indicating significance compared to the control. UAE—extract obtained by ultrasound-assisted extraction.

In the following experimental set, contractions induced by histamine were statistically significantly reduced by the UAE. The maximum contraction of histamine (100%), at 300 nM, was reduced by the extract to 69.50 ± 3.78% with the concentration of 1.5 mg/mL and to 51.72 ± 1.57% with the concentration of 5 mg/mL. The EC_50_ of histamine was modified by both of the extract’s concentrations, from 7.93·10^−11^ ± 2.01·10^−12^ to 2.02·10^−5^ ± 2.00·10^−6^ nM and 6.95 ± 0.11 nM, respectively (Figure 4).

#### 2.2.4. Effects of the *G. lutea* Extract on Calcium-Chloride-Induced Ileum Contractions

**Figure 5:**
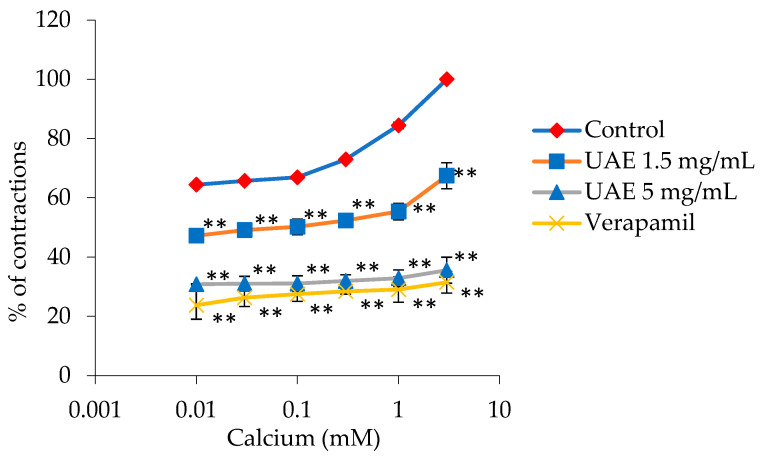
Relaxant effects of the *G. lutea* L. extracts, UAE (1.5 mg/mL and 5 mg/mL), and verapamil (0.3 μM) on the CaCl_2_-induced contractions of the isolated rat’s ileum. Each data point represents the mean value of the response, accompanied by the SD of six segments. Statistical analysis was carried out using Student’s *t*-test, with ** *p* < 0.01 indicating significance compared to the control. UAE—extract obtained by ultrasound-assisted extraction.

Figure 5 illustrates the inhibition of ileum contractions caused by calcium ion action (0.01–3 mM) with the UAE *G. lutea* extract. At an extract concentration of 1.5 mg/mL, maximum ileum contractions caused by calcium ions (100%) were reduced to 67.48 ± 4.38%, and with an extract concentration of 5 mg/mL, they were reduced to 35.59 ± 0.80%. The extract was able to modify the EC_50_ value of the control (2.33·10^−3^ ± 1.0·10^−4^ mM) to 0.16 ± 0.01 mM and 12.26 ± 0.88 mM, respectively. Verapamil, a calcium channel antagonist, reduced contractions to 31.49 ± 3.65% with an applied dose of 0.3 μM (Figure 5), and EC_50_ of 12.73 ± 0.53 mM.

#### 2.2.5. Effects of the *G. lutea* Extract on Potassium-Chloride-Induced Ileum Contractions

Similar to spontaneous contractions, the extract was also inhibitory on the contractions induced by the application of a KCl solution (80 mM), but to a more significant degree. The relaxation of the smooth ileum musculature was dose-dependent, with maximum extract concentrations of 5 mg/mL reducing induced contractions to 61.39 ± 4.84% with an EC_50_ of 47.77 ± 3.54 mg/mL. Verapamil, used as a control in this study, at a maximum concentration of 0.005 mg/mL, reduced the contractions to 38.06% with an EC_50_ of 1.64·10^−1^ ± 3.01·10^−5^ mg/mL.

**Figure 6:**
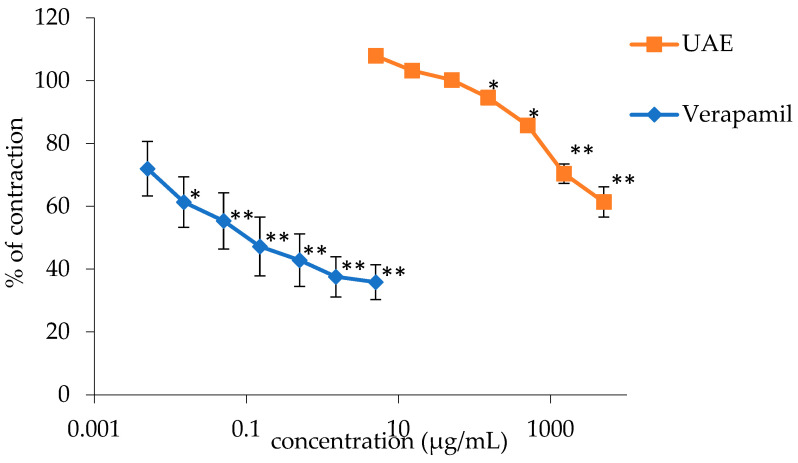
Relaxant effects of the *G. lutea* L. extracts (UAE) and papaverine on KCl-induced rat’s ileum contractions. Each data point represents the mean value of contractions in relation to contractions observed in the Tyrode solution (control), accompanied by the SD of six segments. Statistical analysis was conducted using Student’s *t*-test, with * *p* < 0.05 and ** *p* < 0.01 denoting significance compared to the Tyrode solution. UAE—extract obtained by ultrasound-assisted extraction.

The figure represents the dose-dependent spasmolytic activities of the extract and verapamil (Figure 6).

#### 2.2.6. Effects of the *G. lutea* Extract on the Contractions Induced by an Agonist of L-Type Ca^2+^ Channels (Bay K8644)

**Figure 7:**
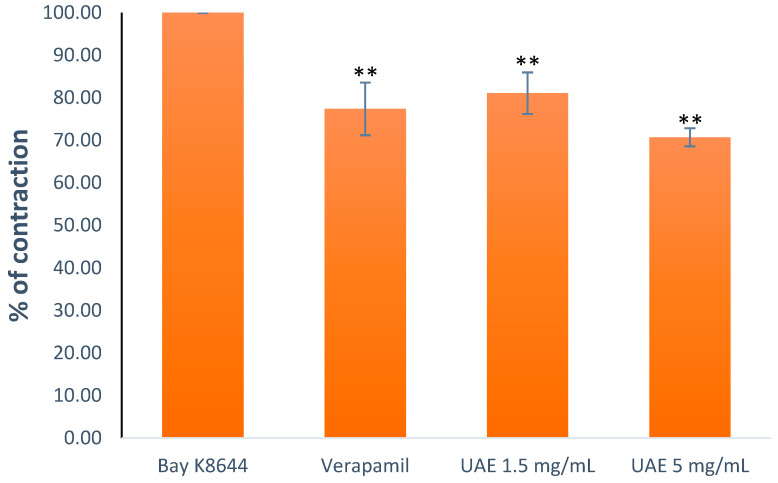
Relaxant effects of the *G. lutea* UAE (1.5 mg/mL and 5 mg/mL) and verapamil (10^−6^ M) on the contractions caused by Bay K8644. Each data point represents the mean value of the response, accompanied by the SD of six segments. Statistical analysis was carried out using Student’s *t*-test, with ** *p* < 0.01 indicating significance compared to the Bay K8644. UAE—extract obtained by ultrasound-assisted extraction.

A rat ileum, pre-incubated with the extract (0.5 and 1.5 mg/mL) or the positive control verapamil (10^−6^ M), was treated with Bay K8644, and the results were compared with a non-incubated test batch influenced by Bay K8644 alone (10^−6^ M). The extract modified the contractions to 81.10 ± 4.87% with the 1.5 mg/mL concentration and 70.73 ± 2.12% with the 5 mg/mL concentration while verapamil, the positive control, reduced the contractions to 77.40 ± 6.19% (Figure 7). 

#### 2.2.7. Role of NO and cGMP on the Effect of *G. lutea* Extract on Spontaneous Contractions (L-NAME and ODQ)

**Figure 8:**
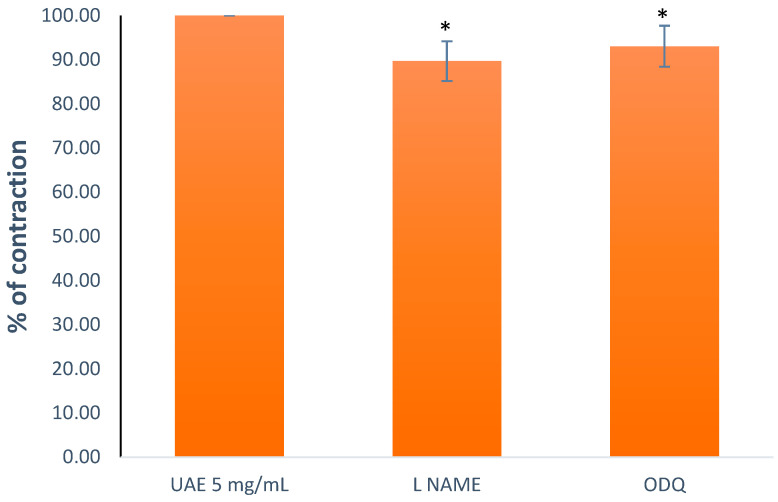
The effect of the *G. lutea* UAE (5 mg/mL, 100%) on the contractions modified by L-NAME and ODQ. Each data point represents the mean value of the response, accompanied by the SD of six segments. Statistical analysis was carried out using Student’s *t*-test, with * *p* < 0.05 indicating significance compared to UAE 5 mg/mL. UAE—extract obtained by ultrasound-assisted extraction.

To investigate the role of NO and cGMP, we used two compounds, L-NAME (10 μM), a suppressor of NOS, and ODQ (1 μM), a potent and specific suppressor of soluble guanylyl cyclase. Determining the effect of the substances on the extract’s (5 mg/mL) effect was carried out by pre-treating the ileum segments 20 min before the addition of the extract with the aforementioned compounds. The pre-treatment with both of the compounds diminished the impact of the *G. lutea* UAE on the contractions (Figure 8), with L-NAME reducing the extract’s effect on the spontaneous contractions to 89.67 ± 4.48% and ODQ reducing them to 93.03 ± 4.65%. The substances facilitating the NO/cGMP pathway did not demonstrate any inherent effect on spontaneous motility.

#### 2.2.8. Role of K^+^ Channels in Effect of *G. lutea* Extract on Spontaneous Contractions (Apamin, TRAM-34, Charybdotoxin, Glibenclamide, and Quinine)

**Figure 9:**
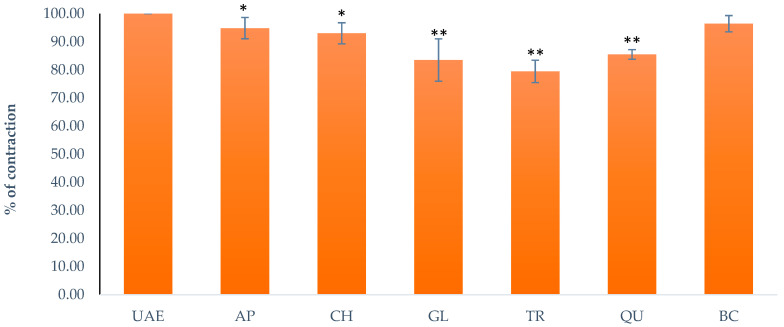
The effect of the *G. lutea* UAE (5 mg/mL, 100%) on the contractions modified by Apamin (AP), Charybdotoxin (CH), Glibenclamide (GL), TRAM-34 (TR), Quinine (QU), and BaCl2 (BC). Each data point represents the mean value of the response, accompanied by the SD of six segments. Statistical analysis was carried out using Student’s *t*-test, with * *p* < 0.05 and ** *p* < 0.01 indicating significance compared to the UAE 5 mg/mL. UAE—extract obtained by ultrasound-assisted extraction.

To study the effects on K^+^ channels, different compounds that affect the various K^+^ pathways were applied as a pre-treatment to the isolated ileum. After 20 min, the extract (5 mg/mL) was applied and the results were measured. Apamin (1 μM), a selective voltage-activated K^+^ channel and small-conductance Ca^2+^ inhibitor, produced a reduction of the contractions to 94.85 ± 3.79%. BaCl_2_ (0.9 mM), an inhibitor of the inward rectifier K^+^ channel, produced a reduction of the contractions to 96.43 ± 2.89%. Charybdotoxin (0.01 μM), a specific inhibitor of intermediate- and large-conductance Ca^2+^-activated K^+^ channels, produced a reduction of the contractions to 93.03 ± 3.72%. Glibenclamide (10 M), an inhibitor of ATP-sensitive K^+^ channels, produced a reduction of the contractions to 83.52 ± 7.51%. TRAM-34 (1 μM), a selective inhibitor of intermediate-conductance Ca^2+^-activated K^+^ channels, produced a reduction of the contractions to 79.48 ± 3.97%. Quinine (10 μM), an inhibitor of voltage-sensitive K^+^ channels, produced a reduction of the contractions to 85.54 ± 1.71% (Figure 9).

A graphical representation of the tested spasmolytic effects of *G. lutea* root extracts has been presented in the supplementary files (Figure S8). 

## 3. Discussion

The reported chemical composition of UAE, HAE, and percolation extracts (Table 1) is consistent with the reports of Jiang et al. [7] and Ponticelli et al. [21], who also identified secoiridoids, xanthones, and flavonoids as the main bioactive components in plant species of the genus *Gentiana*. The phytochemical profiles of all observed extracts were similar, with gentiopicroside as the principal compound, followed by loganic acid, isogentisin, sweroside, swertiamarin, and trace amounts of isovitexin, respectively. These results are in good agreement with those of Mustafa et al. [22], where the analysis of twenty samples of cultivated, wild, and commercial yellow Gentian revealed that gentiopicroside is the most dominant compound, followed by loganic acid, isogentisin, sweroside, swertiamarin, and finally amarogentin. In the mentioned study, it is highlighted that the variations in the levels of bioactive components depend on factors such as geographical origin, developmental stage, and processing of harvested plant materials like handling, drying, and extraction conditions. For instance, bitter secoiridoid content significantly varies based on the plant’s age, while xanthone content is not significantly age-dependent but instead linked to the vegetation phase. Thus, isogentisin reaches its peak during the flowering stage and is at its lowest during the non-vegetative period [22].

The highest extraction efficiency for all individual compounds was observed with UAE, except for gentiopicroside, where a slightly better efficiency was noted in the case of percolation. The effectiveness of UAE can be attributed to distinct extraction mechanisms when compared to conventional methods. Namely, the root of *G. lutea* is a rich source of polysaccharides (pectin and inulin), which, within the plant matrix, act as binders for extractable compounds, thereby reducing their transfer into the liquid phase during extraction [23]. UAE generates acoustic cavitation and a strong shear force that facilitates matrix disruption (sonoporation), enhances the desorption of extractable compounds, and enhances their solid–liquid mass transfer [23,24]. Furthermore, UAE provides a significant reduction in extraction time.

Regarding extract fractionation, the most pronounced separation was recorded in the case of isogentisin fractionation with organic solvents, probably due to its low polarity. This is consistent with previous findings that isogentisin, as a less polar compound, demonstrates higher extraction efficiency at elevated concentrations of ethanol during heat-assisted extraction with a hydroethanolic mixture in comparison to more polar secoiridoids and flavonoids extracted from the underground parts of *Gentiana asclepiadea* [25]. The flavonoid isovitexin was only quantified in the n-butanol fraction (5.92 ± 0.24 mg/g DW), while in other fractions and crude extracts, it was either present in trace amounts or undetected. A similar distribution pattern of flavonoids of *Sideritis raeseri* subsp. *raeseri* hydroethanolic extract was reported by Krgović et al. [26], where the n-butanol fraction showed the highest concentration of hypolaetin and isoscutellarein derivatives. Overall, no single fraction is optimal for separating all individual compounds simultaneously, but the choice depends on specific target compounds of interest.

The *G. lutea* extracts showed a varying effect on the spontaneous contractility of the rat ileum, with the UAE being the most effective, showing a significant effect compared to the positive control. The spasmolytic effect seems to corroborate the data found in other plants in the Gentianaceae family [19,27]. Common traits of the family are the bitter substances, such as gentiopicroside, swertiamarin, and sweroside, that are presumed to be the cause of this activity, as well as some less prevalent compounds that add to the effect [7,19].

Due to its effectiveness, the UAE was fractionized and tested on spontaneous contractility, which showed that the petroleum ether and ethyl acetate fractions provided the best responses. Referencing the chemical composition of these fractions provided insight into the specific concentrations of available compounds in the extracts; following this, we come to the conclusion that isogentisin, a xanthone, is the prevalent compound found in both of these fractions. While, to the authors’ knowledge, there are no specific studies on the spasmolytic effect of isogentisin, members of the same compound class have been shown to exhibit anti-diarrheal effects [28]. From this, we can draw a conclusion that isogentisin is a main contributing factor in the spasmolytic effect of the gentian extract. 

Swertiamarin, a secoiridoid heteroside, was found in low concentrations in the base extracts and was not present in the most effective fractions, with regard to the spasmolytic effect. Gentiopicroside, a member of the same compound group, was present in high concentrations in the base extracts. However, it was lower in concentration when fractions were examined, in the fractions that had high spasmolytic effects, compared to its high concentration in those that had no effect. This shows that secoiridoid heterosides, even if found in large doses in the extract, have no apparent effect on the extracts’ inhibitory effect on the ileum spontaneous contractions.

Compared to the better-performing fraction (petroleum ether), from a spasmolytic effect standpoint, the ethyl acetate fraction contains a higher concentration of isogentisin, and the concentration of loganic acid, sweroside, and gentiopicroside is also higher. These results show that one or more of these compounds may be responsible for the lowering of the isogentisin effect and the overall spontaneous contraction spasmolytic effect.

On another note, a study [19] focusing on gentiopicroside activity on ileum smooth muscle had shown gentiopicroside’s ability to reduce the spontaneous contractions with an EC_50_ of 2.80 ± 0.71 µg/mL. This dose had been used to show gentiopicroside’s ability to inhibit acetylcholine-, histamine-, BaCl_2_-, and KCl-induced contractions [19]. These findings correlate to the effect of the *G. lutea* extract itself on these types of contractions, seeing as it was a prevalent compound in the tested extract. The herbal ethanolic extract of *Gentianopsis paludosa*, also a member of the Gentianaceae family, has been found to exhibit inhibitory effects on rat duodenum contractions caused by acetylcholine and KCl, as well as those caused by CaCl_2_ [28]. Similar findings have shown the aqueous extract of the aerial parts of *Centaurium erythraea*, Gentianaceae, to pose inhibitory effects on spontaneous and acetylcholine-, KCl-, and CaCl_2_-induced rabbit jejunum contractions [29]. 

According to the data gathered, the extract showed a great influence on calcium-mediated contractions, seeing as it produced a good effect with KCl, whose mechanism stems from voltage-operated Ca^2+^ channel activation [30]. The extract showed a better effect with the L-type Ca^2+^ channel agonist, Bay K8644, and even better effect regarding the experiments on contractions caused with CaCl_2_ [31]. Recently, an herb mixture containing the *G. lutea* plant was found to exhibit spasmolytic effects through the inhibition of Ca^2+^ pathways [32]. This, combined with the previously stated works on plants from this family [27], can corroborate the results we have shown. 

The role of NO in the effect of the extract was shown to be relatively small. This was found by reducing extract-stimulated contractions with a suppressor of NOS, L-NAME, and its subsequent secondary messenger cGMP, by reducing extract-stimulated contractions with a potent and specific suppressor of soluble guanylyl cyclase, ODQ. L-NAME produced slightly better results than ODQ but was deemed not statistically relevant as the mechanism of action of the *G. lutea* extract. This is contrary to the findings in the aforementioned study with *C. erythraea* extract, which found that this pathway was one of the key mechanisms for its spasmolytic effect.

In the case of potassium-mediated channels, our results show that the selective inhibitor of intermediate conductance Ca^2+^-activated K^+^ channels TRAM-34, the ATP-sensitive K^+^ channel inhibitor glibenclamide, and the voltage-sensitive K^+^ channel inhibitor quinine significantly reduced the spasmolytic effects of *G. lutea* extract on the spontaneous contractions of isolated rat ileum segments. However, the relaxant potential of *G. lutea* extract was not statistically significantly modified by the specific inhibitor of intermediate- and large-conductance Ca^2+^-activated K^+^ channels charybdotoxin, the selective small-conductance Ca^2+^ and voltage-activated K^+^ channel inhibitor apamin, and the inward rectifier K^+^ channel inhibitor BaCl_2_.

Potassium channels play a significant role in regulating the contractility of intestinal musculature activation by causing a significant efflux of K^+^ ions from smooth muscle cells, leading to an increased negativity of their membrane potential. This resulting hyperpolarization inhibits voltage-dependent Ca^2+^ channels, reduces the concentration of Ca^2+^ ions in the cytoplasm of smooth muscle cells, and causes relaxation of isolated ileum [31,33].

The obtained results show that potassium channel blockers TRAM-34, glibenclamide, and quinine significantly reduce the spasmolytic effects of *G. lutea* extract on the smooth musculature of rat ileum, indicating that the relaxant activity of the extract is primarily mediated through intermediate-conductance Ca^2+^-activated K^+^ channels, ATP-sensitive K^+^ channels, and voltage-sensitive K^+^ channels. However, the statistically non-significant effects of charybdotoxin, apamin, and BaCl_2_ on blocking the spasmolytic effects of the extract indicate that intermediate- and large-conductance Ca^2+^-activated K^+^ channels, small-conductance Ca^2+^ channels, voltage-activated K^+^ channels, and inward rectifier K^+^ channels do not have a significant role in the inhibitory activity of *G. lutea* extract.

## 4. Materials and Methods

### 4.1. Plant Materials 

Underground parts of *G. lutea* (Gentian roots) were sourced from a commercial cultivator, a registered agricultural household located on Mount Tara in southwest Serbia, during the 2022 season. The plant material has been authenticated by the quality control sector of the Institute for Medicinal Plant Research “Dr. Josif Pančić”, Belgrade (product serial number: 29180922). Gentian roots were air-dried, and before the extraction process, dried root pieces were ground using a laboratory mill. Particles ranging from 0.75 to 2 mm in size were separated using laboratory sieves according to the Yugoslav Pharmacopoeia (Ph. Yug. V) and were subsequently used for the extraction.

### 4.2. Extraction Procedure 

Extraction of *G. lutea* underground parts was performed using three different extraction techniques: ultrasound-assisted extraction, heat-assisted extraction, and percolation. The said extracts were labeled UAE, HAE, and PE, respectively. The first extract was obtained using a previously optimized and published method for UAE [23] with slight modifications. The following conditions were applied: extraction temperature of 80 °C, time of 30 min, solid-to-solvent ratio of 1:20 g/mL, and ethanol concentration of 50%. The second extract was obtained using a previously optimized and published method for HAE [14] with slight modifications. The following conditions were applied: extraction temperature of 65 °C, time of 130 min, solid-to-solvent ratio of 1:20 g/mL, and ethanol concentration of 50%. The third extract was prepared using a triple percolation technique with 50% ethanol and drug to extract ratio of 1:2 g/mL. After filtration, all of the obtained extracts were evaporated to dryness on a vacuum evaporator (Buchi rotavapor R-114) and stored at 4 °C for further chemical analysis.

### 4.3. Fractionation of the Selected Extract

Due to the highest content of bioactive compounds, the extract obtained using UAE was further subjected to fractionation. Prepared dry extract (35 g) was dissolved in 100 mL of distilled water and then successively re-extracted with organic solvents in a separatory funnel (6 × 50 mL of petroleum ether, 3 × 100 mL of ethyl acetate, and 3 × 50 mL of n-butanol). The residual water phase was considered as a water fraction. After evaporation of the solvent using a vacuum evaporator, dry residues were obtained and further analyzed. 

### 4.4. HPLC Analysis

HPLC analysis was performed using a previously described procedure [23]. An Agilent 1260 RR HPLC instrument (Agilent, Waldbronn, Germany) equipped with a diode-array detector (working range 190–550 nm) was utilized. Samples were separated on a reverse-phase Zorbax SB-C18 (Agilent) analytical column (150 mm × 4.6 mm i.d.; 5 µm particle size). The temperature of the column was held at 25 °C during the entire analysis. The mobile phase A was 1% *v*/*v* solution of orthophosphoric acid in water, mobile phase B was acetonitrile. The following gradient program was applied: 98–90% A, 0–5 min; 90% A, 5–10 min; 90–85% A, 10–18 min; 85–70% A, 18–23 min; 70–40% A, 23–27 min; 40–0% A, 27–31 min; post time 5 min, flow at 1 mL/min; selected detection wavelengths were 260 and 320 nm. Identification of the compounds was performed by linking their absorbance spectra and retention time with those from authentic substances. Quantification of the compounds was performed using calibration curves for each of them. The results are presented as micrograms per gram of dry weight (μg/g dw) for solid samples.

### 4.5. Experimental Animal Housing

The experimental procedures adhered to the guidelines outlined in the European Directive 2010/63/EU for animal experimentation. Approval for these procedures was obtained from the Veterinary Directorate of the Republic of Serbia Ministry of Agriculture, Forestry and Water Management, with the assigned decision number 323-07-08991/2022-05. Male *Wistar* albino rats, aged 10–12 weeks, and weighing between 200 and 250 g, were procured from the Vivarium of the Research Centre for Biomedicine of the Faculty of Medicine, University of Niš. To ensure acclimatization, the animals were individually housed one week before the commencement of experiments. Throughout this duration, they were housed in stainless steel cages within standard laboratory settings, ensuring a controlled room temperature between 20 and 24 °C and adhering to a 12 h light–dark cycle. All animals were granted unrestricted access to food and water throughout the experimental period, except during the 24 h preceding the experiments when access to food was restricted.

### 4.6. Methodology for Isolated Rat Ileum Contractions

#### 4.6.1. The Isolation and Placement of Rat Ileum

The rat is placed in a closed chamber and exposed to ether vapors at the beginning of the experiment. After a successful anesthesia effect, the chest is opened, and the aorta is cut. From there on, segments of the ileum in 2 cm strips are cut and isolated from the mesentery. The prepared segments of the ileum are then placed in a bath containing 20 mL of physiological solution for isolated intestines (Tyrode’s solution), with the temperature being 37 °C, and maintained under a continuous flow of a blend of oxygen and carbon dioxide (95% and 5%). The composition of the Tyrode’s solution includes the following constituents: 150 mM NaCl, 2.7 mM KCl, 2 mM MgCl_2_, 12 mM NaHCO_3_, 0.4 mM NaH_2_PO_4_, 1.8 mM CaCl_2_, and 5.5 mM glucose. Half an hour before the start of the experiment, the ileum is stabilized in the bath [34].

Alterations in small intestine contractility were monitored using a transducer (Transducer–TSZ-04-E, Experimetria Ltd., Budapest, Hungary), and the acquired data were processed utilizing the SPEL Advanced ISOSYS Data Acquisition System software.

#### 4.6.2. The Impact of *G. lutea* Extracts on Spontaneous Contractions of Rat Ileum

Three *G. lutea* extracts, UAE, HAE, and PE, were tested for their effect on spontaneous contractions of isolated ileum first. The extract solutions were added after an adaptation period in cumulative doses (0.005, 0.015, 0.05, 0.15, 0.5, 1.5, and 5 mg/mL), and a dose-dependent response was recorded. The results were calculated as the difference between the areas under the curve before and after adding the samples.

The spasmolytic effect, at every concentration of the extract, was quantified as a percentage in relation to the initial spontaneous activity of the isolated intestine in the absence of the tested extract [34].

Papaverine, a non-specific miorelaxant with a high spasmolytic activity, was used as a positive control in concentrations ranging from 0.01 to 3 μg/mL.

The same methodology was used on the UAE fractions, with the exception of the cumulative dose being from 0.005–1.5 mg/mL.

#### 4.6.3. The Effects of the *G. lutea* Extract on Acetylcholine-Induced Contractions of Rat Ileum

For evaluating the impact of the UAE on acetylcholine-induced contractions, a concentration-dependent contraction control curve was generated by stimulating ileum contractions with cumulative additions of increasing concentrations of acetylcholine (5, 15, 50, 150, 500, and 1500 nM) following an adaptation period. Subsequently, the ileum preparations were rinsed with Tyrode’s solution to restore stable spontaneous contractions.

After washing the tested ileum, a test solution containing the extract at a concentration of 1.5 mg/mL was introduced into the organ bath. Following a 5 min interval, the series was reiterated with the same acetylcholine concentrations, establishing a new curve to depict the contractile effect of acetylcholine in the presence of the extract.

Ileum preparations were again washed with Tyrode’s solution to re-establish spontaneous contractions and promptly tested with a sample of the same extract with a concentration of 5 mg/mL in the same way as above.

The spasmolytic effect of the extracts is presented through a series of curves that show the contractile effect of acetylcholine (%) in the presence of the tested extracts and is compared with the effect of acetylcholine alone, i.e., in the absence of the extract [34].

For the purposes of positive control, atropine (140 nM), a non-selective blocker of muscarinic receptors, was used.

#### 4.6.4. The Effects of the *G. lutea* Extract on Histamine-Induced Contractions of Rat Ileum

In this series of experiments, a 30 min adaptation period was established before inducing contractions of ileum preparations, after which increasing concentrations of histamine solution (1, 3, 10, 30, 100, and 300 nM) were introduced to the organ bath to induce ileum contractions. A control curve was formed from the dependence of the obtained contractions (%) on the concentrations of histamine solution.

To restore spontaneous contractions, the ileum preparations were rinsed with Tyrode’s solution following the establishment of the control curve. Subsequently, the UAE sample (1.5 mg/mL) was introduced into the organ bath. After a 5 min interval, the series was replicated using identical concentrations of the histamine solution (1–300 nM). The same procedure was conducted with the UAE sample at a concentration of 5 mg/mL.

To assess the spasmolytic effect of the extract, curves of the contractile effect of histamine (%) in the presence of the extract at a concentration of 1.5 mg/mL and 5 mg/mL and in its absence were constructed [34].

#### 4.6.5. The Role of Calcium Channels in the Effect of the *G. lutea* Extract

To better understand the mechanism of *G. lutea* UAE effects, the extract efficiency was compared to known activators of those receptors.

The effects of the *G. lutea* extract on calcium-chloride-induced contractions of rat ileum were tested. The ileum preparations underwent an adaptation period in a calcium-free solution for this experimental setup. Once stable spontaneous contractions were established, increasing concentrations of CaCl_2_ solution (0.01, 0.03, 0.1, 0.3, 1, and 3 mM) were introduced into the organ bath, and the ensuing contractions were recorded. A control curve depicting concentration-dependent contractions was then constructed based on the acquired results. Subsequently, the ileum preparation was washed until stable spontaneous contractions were restored.

Following this, the UAE solution with a concentration of 1.5 mg/mL was added to the organ bath, and after a five-minute interval, the same increasing concentrations of CaCl_2_ were applied. The same protocol was repeated with an extract solution concentration of 5 mg/mL. Curves illustrating the contractile effect of CaCl_2_ in the presence of the tested extract at concentrations of 1.5 mg/mL and 5 mg/mL were generated. Verapamil, administered at a dose of 0.3 µmol/L, served as the standard for comparison.

The spasmolytic effects of the tested extract are elucidated through a series of curves showcasing the contractile effect of CaCl_2_ (%) observed in the presence of the tested samples. These effects are juxtaposed with the outcomes of the control series, where only CaCl_2_ was applied, indicating the absence of the extract [34].

The impact of the *G. lutea* extract on potassium-chloride-induced contractions of rat ileum was tested by adding a KCl solution (80 mM) to the ileum after an adaptation period to induce contractions. Those tonic contractions were then inhibited by the cumulative addition of UAE solutions in concentrations ranging from 0.005 to 5 mg/mL at 15 min intervals. The spasmolytic impact of the extracts was computed by assessing the variances in the areas under the curve compared to the baseline, expressed as a percentage indicative of the inhibition of the KCl effect [34].

As a positive control for this experiment, verapamil, a calcium channel antagonist, was used in concentrations ranging from 0.015 to 1.5 μg/mL.

The impact of the *G. lutea* extract on the agonist of L-type Ca^2+^ channels (Bay K8644)-induced contractions of rat ileum was examined using the following procedure: for 15 min, the ileum segments were incubated with the extract (1.5 and 5 mg/mL) and verapamil (10^−6^ M) before the addition of Bay K8644. The contractile response to Bay K8644 obtained in the presence of *G. lutea* extract or verapamil was compared with the response obtained by Bay K8644 alone (control, 100%) [31].

#### 4.6.6. The Role of NO and cGMP in the Effect of the *G. lutea* Extract

In this part of the experiment, Nω-Nitro-L-arginine methyl ester (L-NAME), an inhibitor of NO synthase (NOS), and 1H-[1,2,4]oxadiazolo[4,3-a]quinoxalin-1-one (ODQ), a potent and selective inhibitor of soluble guanylyl cyclase, were applied. The application was carried out by pre-treating ileum segments with 10 µM L-NAME and 1 µM ODQ separately, 20 min before the extract was administered, to explore the involvement of the nitric oxide (NO)/GMP pathways in the relaxation effect triggered by UAE (5 mg/mL) in spontaneous contractions.

Results were compared with those achieved by the extract alone on the same organ tissue [31].

#### 4.6.7. The Role of K^+^ Channels in the Effect of the *G. lutea* Extract

For this experimental set, various substances were used: apamin, a selective small-conductance Ca^2+^- and voltage-activated K^+^ channel (SKCa) inhibitor, in a 1 µM dose; BaCl_2_, an inhibitor of the inward rectifier K^+^ channel (KIR), in a 0,9 mM dose; charybdotoxin, a specific inhibitor of intermediate- and large-conductance Ca^2+^-activated K^+^ channels, in a 0.01 µM dose; glibenclamide, an inhibitor of ATP-sensitive K^+^ channel (KATP), in a 10 µM dose; 1-[(2-chlorophenyl) diphenylmethyl]-1H-pyrazole (TRAM-34), a selective inhibitor of intermediate conductance Ca^2+^-activated K^+^ channels (IKCa), in a 1 µM dose; and quinine, an inhibitor of voltage-sensitive K^+^ channels, in a 10 µM dose.

In order to induce contractions in the ileum segments, the aforementioned substances, each with their own concentration, were separately added 20 min before the addition of the extract in a 5 mg/mL dose in order to see the effect those substances had on the extract contraction.

Results were then compared with those achieved by the extract alone on the same organ tissue [31].

### 4.7. Statistical Analysis

The obtained results are shown as average figures from three or six measurements, for either chemical composition or spasmolytic examinations, ± the standard deviation. The EC_50_ values, depicting the concentrations causing half of the maximum reaction, were acquired via regression analysis. Either Student’s *t*-test or one-way ANOVA with Duncan’s subsequent test was applied to ascertain significant statistical variances amid the averages (*p* < 0.05 or *p* < 0.01). The statistical assessments were conducted utilizing the SPSS 20.0 statistical software (SPSS, Inc., Chicago, IL, USA).

## 5. Conclusions

Our research shows, for the first time, that the *Gentiana lutea* L. root extracts and their fractions exhibit great spasmolytic activities in regard to the rat ileum. The mechanism of this spasmolytic action is mediated primarily through mechanisms that directly activate K^+^ and Ca^2+^ channels. Our results provide new insights that support the traditional use of the *G. lutea* root. The root shows excellent potential in the treatment of spasmodic gastrointestinal ailments, and further studies could be undertaken in the realm of internal motor function preservation drug development and discovery. This paper provides a chance for future works to go into more detail on the qualitative compositions of the plant extract, such as an LC-MS analysis.

## Figures and Tables

**Table 1:** Chemical composition of crude *G. lutea* root extracts prepared by ultrasound-assisted extraction (UAE), heat-assisted extraction (HAE), and percolation (PE), and fractions obtained by liquid/liquid re-extraction of the most effective UAE extract (mg/g DW of extract or fraction).

Sample	Loganic Acid	Sweroside	Swertiamarin	Gentiopicroside	Isogentisin	Isovitexin
	mg/g DW					
UAE	13.70 ± 0.17 ^a^	1.65 ± 0.03 ^a^	1.05 ± 0.03 ^a^	51.78 ± 1.11 ^b^	11.50 ± 0.16 ^a^	tr
HAE	13.66 ± 0.36 ^a^	1.39 ± 0.04 ^b^	0.72 ± 0.02 ^c^	50.33 ± 1.96 ^b^	10.85 ± 0.31 ^b^	tr
PE	12.83 ± 0.22 ^b^	1.16 ± 0.01 ^c^	0.89 ± 0.02 ^b^	62.54 ± 1.23 ^a^	3.00 ± 0.05 ^c^	tr
Petroleum ether fraction	nd	11.08 ± 0.34 ^c^	nd	5.80 ± 0.09 ^c^	262.30 ± 5.03 ^b^	nd
Ethyl acetate fraction	133.22 ± 4.08 ^a^	62.95 ± 1.27 ^a^	nd	23.75 ± 0.85 ^b^	871.58 ± 9.16 ^a^	nd
*n*-Butanol fraction	31.10 ± 1.51 ^b^	23.75 ± 1.08 ^b^	15.54 ± 0.61 ^a^	221.71 ± 8.87 ^a^	145.12 ± 2.78 ^c^	5.92 ± 0.24
Water fraction	9.75 ± 0.41 ^c^	1.00 ± 0.04 ^d^	1.28 ± 0.04 ^b^	16.07 ± 0.51 ^bc^	nd	tr

Mean values ± SD followed by different letters in superscript within the same column indicate significant differences in contents among different crude extracts or different fractions, according to ANOVA with Duncan’s *post hoc* test (*p* < 0.05). tr—detected in traces; nd—not detected.

**Table 2:** The values of EC_50_ and the maximum reductions of spontaneous ileum contraction of the *Gentiana lutea* L. extracts and papaverine.

Samples	Crude Extracts			Fractions of UAE				Positive Control
	UAE	HAE	PE	Petroleum Ether	Ethyl Acetate	n-Butanol	Water	Papaverine
EC_50_ (mg/mL)	2.34 ± 0.15 ^a^	6.82 ± 0.33 ^b^	2.70 ± 0.11 ^c^	0.59 ± 0.01 ^d^	0.65 ± 0.01 ^e^	/	/	3.18 × 10^−2^ ± 0.01 × 10^−2 f^
Maximal reduction of contractions (%)	46.19 ± 4.60 ^a^	29.98 ± 2.56 ^b^	33.69 ± 2.79 ^b^	32.15 ± 2.08 ^b^	28.77 ± 2.62 ^b^	/	/	62.72 ± 2.72 ^c^

UAE, HAE, PE—extract obtained by ultrasound-assisted extraction, heat-assisted extraction, and percolation. The results represent the mean of the six measurements ± the standard deviation (SD). Different lowercase letters in the rows indicate statistically significant differences (Duncan test, *p* < 0.05).

## Data Availability

Data are contained within the article.

## Supplementary Materials

The following supporting information can be downloaded at: https://www.mdpi.com/article/10.3390/plants13030453/s1, Figure S1: HPLC chromatogram of UAE recorded at 260 nm: 1—loganic acid; 2—swertiamarin; 3—gentiopicroside; 4—sweroside; 5—isovitexin; 6—isogentisin; Figure S2: HPLC chromatogram of HAE recorded at 260 nm: 1—loganic acid; 2—swertiamarin; 3—gentiopicroside; 4—sweroside; 5—isovitexin; 6—isogentisin; Figure S3: HPLC chromatogram of PE recorded at 260 nm: 1—loganic acid; 2—swertiamarin; 3—gentiopicroside; 4—sweroside; 5—isovitexin; 6—isogentisin; Figure S4: HPLC chromatogram of petroleum ether fraction recorded at 260 nm: 1—loganic acid; 2—swertiamarin; 3—gentiopicroside; 4—sweroside; 5—isovitexin; 6—isogentisin; Figure S5: HPLC chromatogram of ethyl fraction recorded at 260 nm: 1—loganic acid; 2—swertiamarin; 3—gentiopicroside; 4—sweroside; 5—isovitexin; 6—isogentisin; Figure S6: HPLC chromatogram of *n*-butanol recorded at 260 nm: 1—loganic acid; 2—swertiamarin; 3—gentiopicroside; 4—sweroside; 5—isovitexin; 6—isogentisin; Figure S7: HPLC chromatogram of water fraction recorded at 260 nm: 1—loganic acid; 2—swertiamarin; 3—gentiopicroside; 4—sweroside; 5—isovitexin; 6—isogentisin; Figure S8: A graphical representation of the tested spasmolytic effects of *Gentiana lutea* L. root extracts.
