# Supplementary material for: Spasmolytic Activity of Gentiana lutea L. Root Extracts on the Rat Ileum: Underlying Mechanisms of Action
Source: Plants (Basel). 2024 Feb 4;13(3):453. doi: 10.3390/plants13030453 (PMC10857127; doi:10.3390/plants13030453)
Supplement: Supplementary file 1 [file plants-13-00453-s001.zip › plants-2842892-supplementary.pdf]

Spasmolytic Activity of *Gentiana lutea* L. Root Extracts on the Rat Ileum: Underlying Mechanisms of Action

Nemanja Kitić [1,*], Jelena Živković [2], Katarina Šavikin [2], Milica Randjelović [3], Miloš Jovanović [3], Dušanka Kitić [3,*], Bojana Miladinović [3], Milica Milutinović [3], Nenad Stojiljkovic [4] and Suzana Branković [4]

1 Faculty of Medicine, University of Niš, Research Centre for Biomedicine, Ave. Dr. Zorana Đinđića 81, 18000, Niš, Serbia;
2 Institute for Medicinal Plants Research "Dr. Josif Pančić", Tadeuša Košćuška 1, 11000 Belgrade, Serbia; nemanja.kitic@medfak.ni.ac.rs (J.Ž.); ksavikin@mocbilja.rs (K.Š.)
3 Faculty of Medicine, University of Niš, Department of Pharmacy, Ave. Dr. Zorana Đinđića 81, 18000, Niš, Serbia; milica.randjelovic@medfak.ni.ac.rs (M.R.); milos.jovanovic@medfak.ni.ac.rs (M.J.); bojana.miladinovic@medfak.ni.ac.rs (B.M.); milica.milutinovic@medfak.ni.ac.rs (M.M.)
4 Faculty of Medicine, University of Niš, Department of Physiology, Ave. Dr. Zorana Đinđića 81, 18000, Niš, Serbia; nenstojiljkovic@gmail.com (N.S.); brankovic.suzana@yahoo.com (S.B.)
* Correspondence: nemanja.kitic@medfak.ni.ac.rs (N.K.); dusanka.kitic@medfak.ni.ac.rs (D.K.)

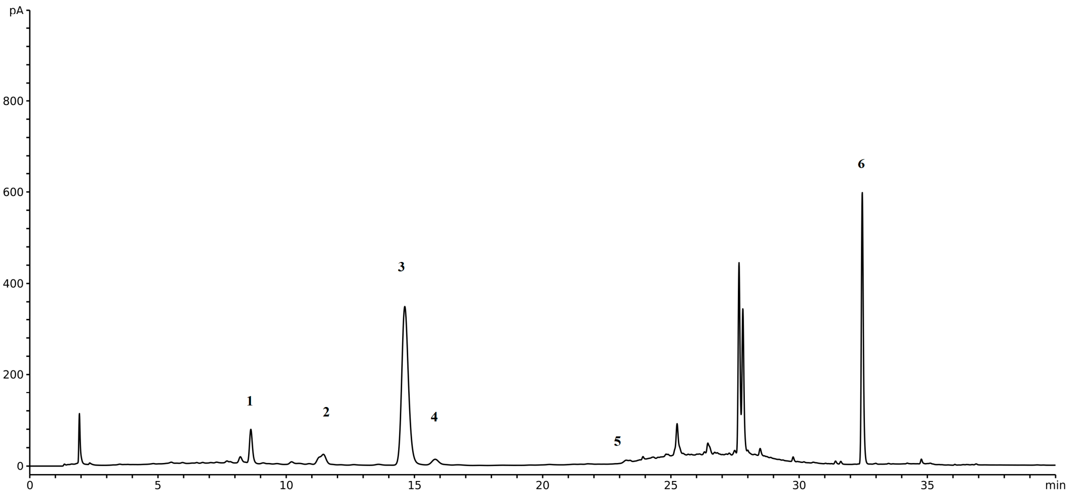

Figure S1. HPLC chromatogram of UAE recorded at 260 nm: 1 - loganic acid; 2 – swertiamarin; 3 – gentiopicroside; 4 – sweroside; 5 – isovitexin; 6 – isogentisin

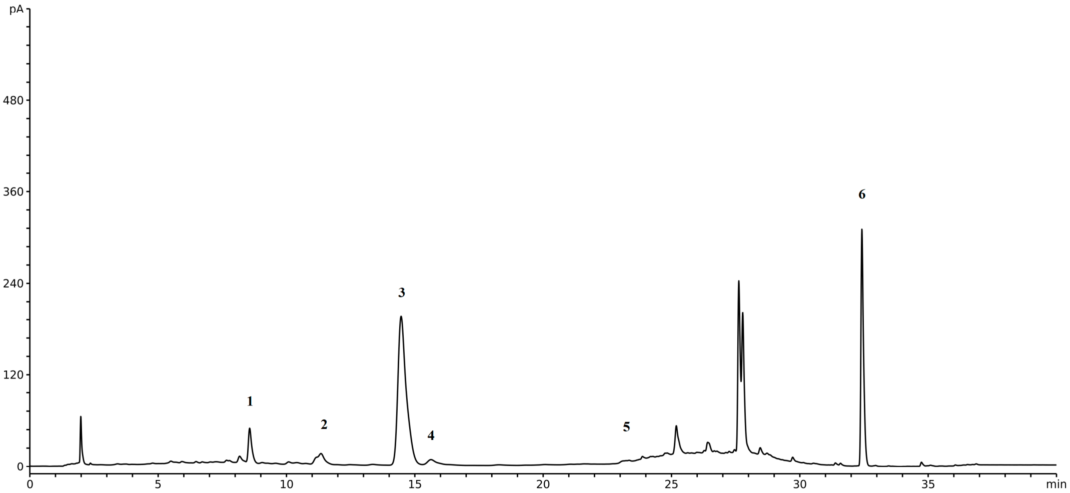

Figure S2. HPLC chromatogram of HAE recorded at 260 nm: 1 - loganic acid; 2 – swertiamarin; 3 – gentiopicroside; 4 – sweroside; 5 – isovitexin; 6 – isogentisin

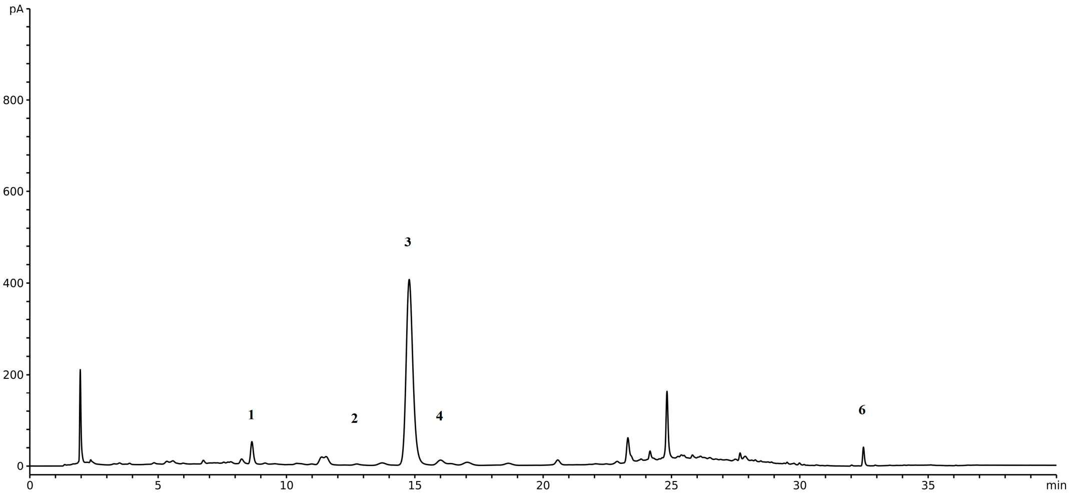

Figure S3. HPLC chromatogram of PE recorded at 260 nm: 1 - loganic acid; 2 – swertiamarin; 3 – gentiopicroside; 4 – sweroside; 5 – isovitexin; 6 – isogentisin

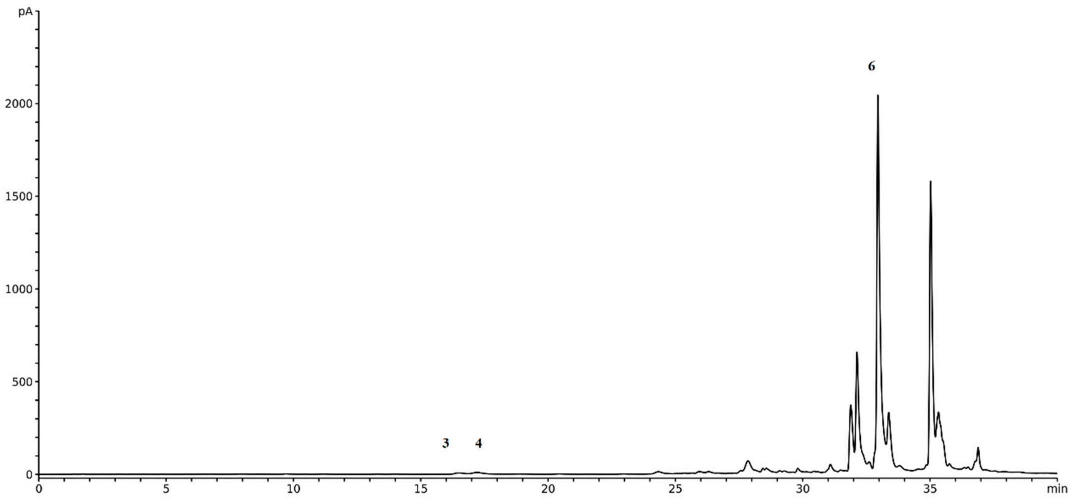

Figure S4. HPLC chromatogram of petroleum ether fraction recorded at 260 nm: 1 - loganic acid; 2 – swertiamarin; 3 – gentiopicroside; 4 – sweroside; 5 – isovitexin; 6 – isogentisin

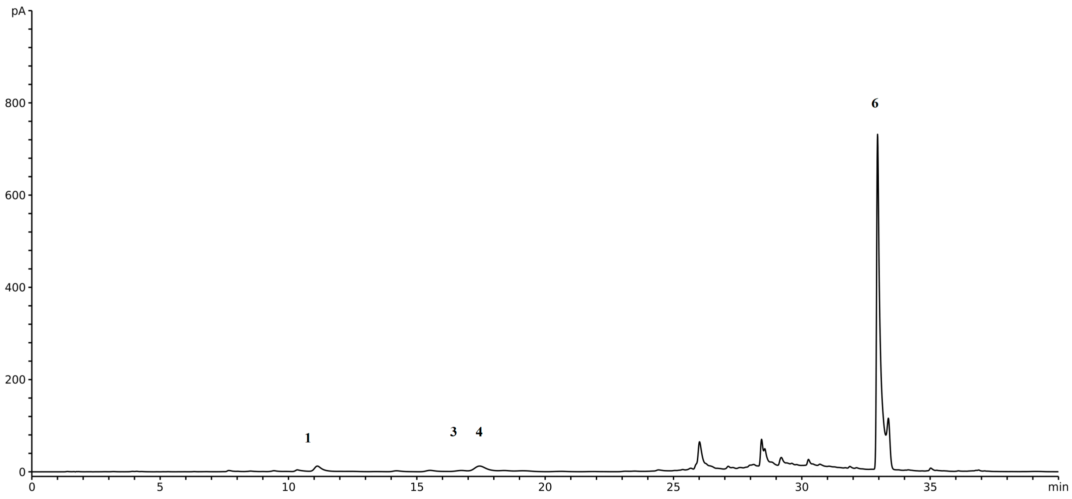

Figure S5. HPLC chromatogram of ethyl fraction recorded at 260 nm: 1 - loganic acid; 2 – swertiamarin; 3 – gentiopicroside; 4 – sweroside; 5 – isovitexin; 6 – isogentisin

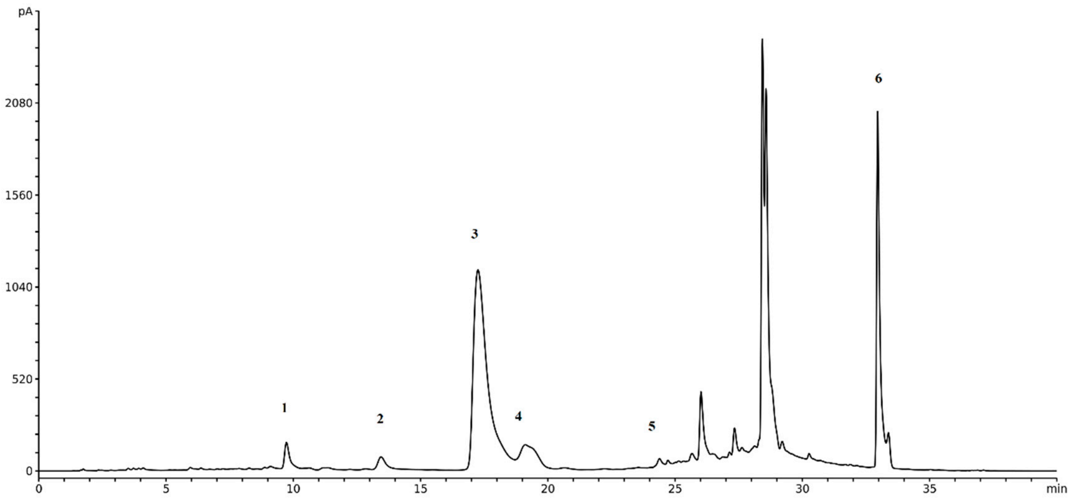

Figure S6. HPLC chromatogram of *n* - butanol recorded at 260 nm: 1 - loganic acid; 2 – swertiamarin; 3 – gentiopicroside; 4 – sweroside; 5 – isovitexin; 6 – isogentisin

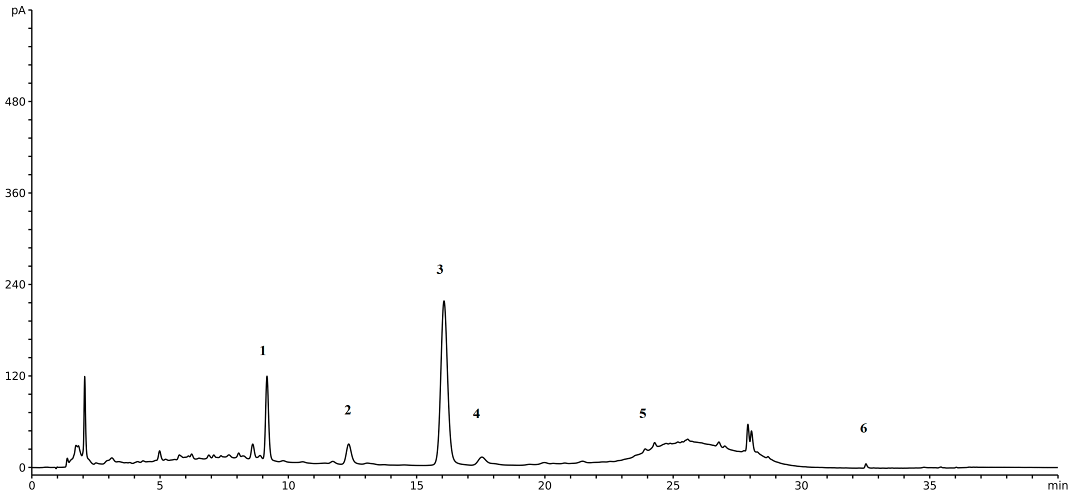

Figure S7. HPLC chromatogram of water fraction recorded at 260 nm: 1 - loganic acid; 2 – swertiamarin; 3 – gentiopicroside; 4 – sweroside; 5 – isovitexin; 6 – isogentisin

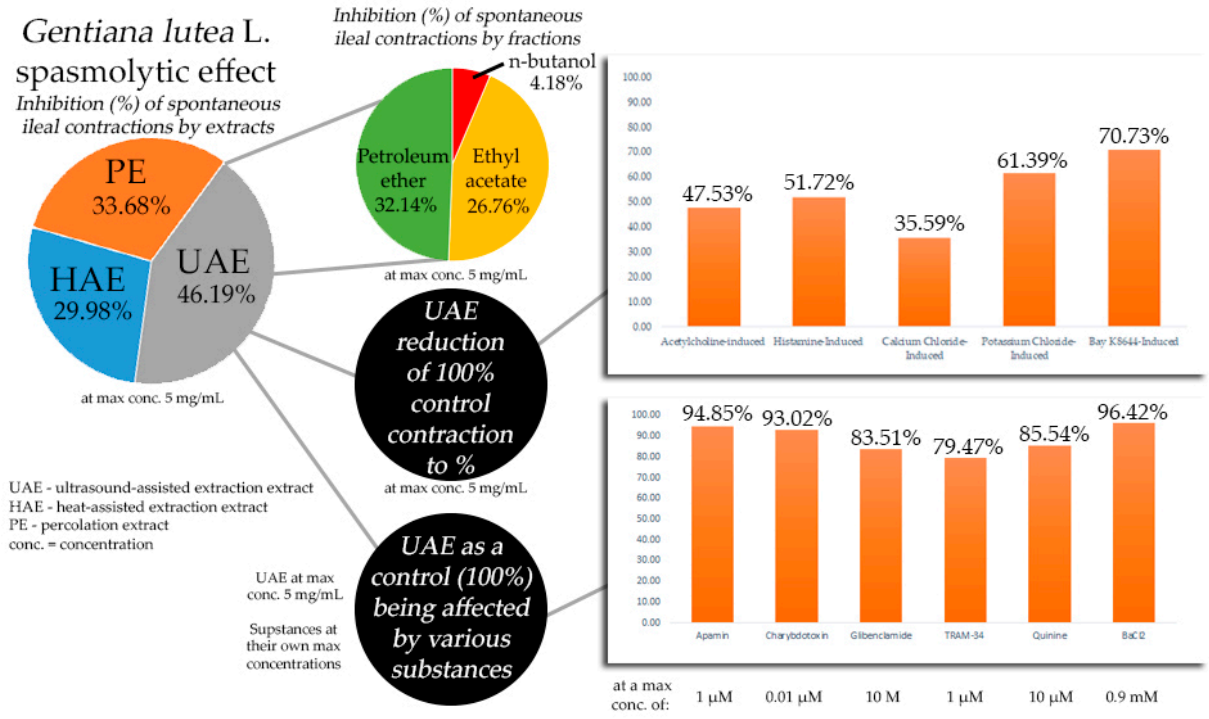

Figure S8. A graphical representation of the tested spasmolytic effects of *Gentiana lutea* L. root extracts
